# Relationship between caries indexes and obesity in a sample of Puerto Rican adolescents

**DOI:** 10.3389/fdmed.2024.1391833

**Published:** 2024-07-26

**Authors:** Lydia M. Lopez del Valle, Mariely Nieves-Plaza, Sona Rivas-Tumanyan, Rosana Hanke-Herrero

**Affiliations:** ^1^ Office of the Assistant Dean of Research, Surgical Sciences Department, University of Puerto Rico School of Dental Medine, San Juan, Puerto Rico; ^2^ Hispanic Alliance for Clinical and Translational Research, Biostatistics, Epidemiology and Research Design (BERD) Core, School of Medicine, Universidad Central del Caribe, San Juan, Puerto Rico; ^3^ Office of Assistant Dean for Research, Department of Surgical Sciences, Biostatistics, Epidemiology and Research Design (BERD) Core, The Hispanic Alliance for Clinical and Translational Research (Alliance), University of Puerto Rico School of Dental Medicine, San Juan, Puerto Rico; ^4^ Graduate Pediatric Dentistry Program, Ecological Sciences Department, University of Puerto Rico School of Dental Medicine, San Juan, Puerto Rico

**Keywords:** caries, obesity, BMI, adolescents, caries indexes

## Abstract

**Background:**

Obesity and caries have become increasingly prevalent. As of yet, research results on the relationship between obesity and caries in children and adolescents have been inconclusive. This study aimed to evaluate the association between caries and obesity in adolescents.

**Methods:**

This was a cross-sectional study of a convenience sample of 187 Puerto Rican adolescents aged 13–19 years, attending, among them, five high schools. Each participant received dental exams for caries using the criteria of the International Caries Detection and Assessment System (ICDAS), had his or her body mass index (BMI) determined, and took a 19-item risk factor questionnaire. The Significant Caries (SiC) index (for DMFT) was calculated to compare obese and healthy adolescents. Statistical analysis was performed using descriptive and inferential statistics.

**Results:**

Nearly 60% of the sample population was female, with a mean age of 15.7 (±1.25). The mean BMI percentile was 72.24 (±28.78); 48.66% of the participants had a healthy weight, 19.79% were overweight, and 30.48% were obese. Dental caries was observed in 68% of the participants; the mean caries index D3-6MFS was 5.17(±5.92) and the mean D3-6MFT was 3.59 (±3.97). No significant differences were found between caries indexes and obesity status (*P* > 0.05). The median (p25, p75) SiC index among overweight/obese adolescents was 9.5 (7, 12), whereas for healthy weight/underweight participants was 7 (5, 8) (*p* < 0.005).

**Conclusions:**

Based on the limited number of participants, no association was found between dental caries (D3-6MFS and D3-6MFT) and obesity (BMI) among adolescents.

## Introduction

Obesity and dental caries, increasingly critical areas of health disparities affecting vulnerable populations, are challenging the effectiveness of the already overburdened public healthcare system of the United States and Puerto Rico (1–3). The research results regarding the relationship between obesity and dental caries in children and adolescents have been mixed and remain inconclusive (4–7). Elias et al. (8) found from 1997 to 2011 reductions in caries prevalence in Puerto Rican 12-year-olds (81%–69%), as well as in mean decayed, missing, and filled teeth (DMFT) scores (3.8–2.5), mean decayed, missing, and filled surfaces (DMFS) scores (6.5–3.9), and mean Significant Caries (SiC) indices (7.3–5.6) (8). There was a more prominent decrease in caries prevalence in private schools compared to public schools; however, there continue to be health disparities in Puerto Rico that are tied to socioeconomic background (8).

Obesity in children and adolescents is a global health problem (1, 9). The prevalence of obesity in children and adolescents has tripled in the last three decades and is greater in Hispanic minorities and black Americans than in any other population. According to World Health Statistics (9), in 2016, 41 million children aged five years or less were overweight or obese. Obesity and overweight have been considered a problem in highly developed countries; however, obesity has begun to impact underdeveloped countries, especially in their urban communities. In 2016, 340 million children and adolescents aged 5–19 years were reported to be overweight or obese. The prevalence of overweight and obesity in children and adolescents (5–19 years of age) has increased disproportionately, rising from 4% in 1975 to 18% in 2016. This increase has been similar in both genders: 18% for girls and 19% for boys (9).

The Centers for Disease Control and Prevention (CDC) reported that the prevalence of obesity in children and adolescents aged 2–19 was 19.3% (about 14.4 million individuals) in 2017–2018; more specifically, the obesity prevalence for 2- to 5-year-olds, 6- to 11-year-olds, and 12- to 19-year-olds were 13.4%, 20.3%, and 21.2%, respectively. Belonging to specific populations also confers risk for childhood obesity, with Hispanic children having a prevalence of 25.6%, non-Hispanic Black children, 24.2%; non-Hispanic White children, 16.1%; and non-Hispanic Asian children, 8.7% (10).

The Puerto Rico Department of Health reported that 39% of 18- to 24-year-olds in Puerto Rico were obese (11). A research study by Garza et al. (12) with youth in Puerto Rico found that 40% of children and adolescents aged 10–19 were overweight or obese.

The association between caries experience and obesity in adolescents has been reported in the literature for the last 20 years, but these reports have been controversial and inconclusive (6, 7). Over the last 7 years, several studies have suggested ***no relationship*** between body mass index (BMI) or obesity status and dental caries in children and adolescents (6, 7, 13–19). Studies involving Hispanics are sparse, with some showing such an association and others not (14, 15). Nevertheless, the literature contains numerous articles reporting a correlation between obesity and adolescent dental caries (20–26). This association is mostly influenced by diet and socioeconomic status. Dental caries and obesity are multifactorial in etiology and are related to social determinants of health and nutritional practices (6, 7).

Systematic reviews and meta-analyses in the literature have reported no conclusive association between caries and overweight/obesity in children (4–7) as detailed in papers from Hayden (5), Carson (7), and others (6). The results reported present a very wide degree of heterogeneity (in patients, interventions, primary outcomes, and types of design), which does not allow for quantitative synthesis of the data (meta-analysis). The outcomes of studies exploring the relationship between BMI and the frequency of dental caries (DMFT/dmft) differ and are inconsistent, with results appearing to be influenced by the non-uniformity of the assessments, settings, measurement procedures of the ages and ethnicities of the participants, and the confounders of dental caries. In a recent narrative review, Alshihri et al. (6) stated: “Both obesity and dental caries are multifactorial diseases, and their association is far more complex than [*sic*] can be explained by a single common risk factor, presenting evidence for the complexity of this association”.

Because studies in Hispanic populations dealing with the association between caries experience and obesity in adolescents are scarce and present inconclusive results, we decided to evaluate the association between caries experience and obesity in a sample of Puerto Rican adolescents and thus contribute to the scientific knowledge on this topic. This study aimed to report the associations of caries prevalence, D3-6MFS, D3-6MFT, SiC index, and BMI in Puerto Rican adolescents in a convenience sample of 187 individuals aged 13–19 years.

## Methods

This cross-sectional study included a convenience sample of 187 adolescents aged 13–19 years enrolled in one private and four public high schools, all in the Municipality of San Juan metropolitan area in Puerto Rico. This study was approved by the University of Puerto Rico Medical Sciences Campus Institutional Review Board (IRB number 0640112). The proposed sample size for the study was 300 adolescents, 75 in each BMI category. The power of the study for 300 adolescents was 0.80. Problems in the recruitment of obese and overweight adolescents arose during the performance of the study because of the participation of these groups. The study ended when 187 students were recruited because the only adolescents who agreed to participate were in the healthy weight category, leaving a small sample size, a limitation of this study. Even with the combined BMI categories, the achieved *post hoc* statistical power for the bivariate analysis was estimated at around 6% for untreated caries prevalence, 18% for caries prevalence, 7% for D3-6S, and 6% for D3-6MFS comparisons.

Scheduled meetings at the participating schools were used to give consent and assent forms to all students who met the age inclusion criteria and their parents. Inclusion criteria were age 13–18 years old, no braces, and all permanent teeth present, except the third molars. Adolescents were excluded if they had braces, were older than 18 years old, or had mixed dentition before the dental examinations of the adolescents. A calibration of the examiners was performed using the International Caries Diagnosis and Assessment System (ICDAS) criteria (27), for both caries and restoration/missingness codes. ICDAS criteria were used in this study because they are the new recommended criteria for evaluating caries, restoration, and missing teeth in children and adolescents in epidemiological studies. The calibration of dental examiners occurred 1 month before the beginning of the study. The calibration activity consisted of 1 week of dental exams in 45 subjects, including children, adolescents, and adults. A gold standard from Tufts U. Intra examiners kappa was obtained and ranged between 0.75 and 0.80. Intra-examiner kappa was obtained after reexamining 20 patients with an intra-kappa of 0.80–0.87.

A one-visit dental examination at the participants' schools was performed by two calibrated dentists. A dental setting with portable dental chairs, lights, dental instruments, and equipment related to BMI determination with a calibrated Tanita to measure height and weight was established at each participating school.

Dental evaluations included an assessment of the plaque index (Silness and Loe criteria) (28), a caries diagnosis, restorations, and missing teeth (using the ICDAS system, starting with criterion 3) to calculate later the D3-6MFT and D3-6MFS indexes (27). Furthermore, a 19-item questionnaire (including questions on smoking habits, exercise practices, and diet) was administered to all the participants. Weight and height measurements were taken for all the participants with a calibrated Tanita, and BMI was calculated based on the CDC standards for sex and age (10). Each participant's BMI was calculated using the age- and sex-specific BMI Percentile Calculator, (10, 29). Based on their BMI percentiles, the participating adolescents were classified as obese (BMI ≥ 95th percentile), overweight (85th percentile < BMI <95th percentile), healthy weight (5th percentile <BMI <85th percentile), or underweight (BMI ≤5th percentile). The presence of caries was defined as showing at least one tooth affected by caries (i.e., a D3-6MFT≥1). In addition, a binary variable of “untreated caries” (yes/no) was considered as one of the main outcomes, defined as having at least one tooth with a decayed surface (D3-6T ≥1). Caries severity/extent was summarized in terms of the number of decayed surfaces and teeth (D3-6S and D3-6T); the number of carious surfaces with ICDAS scores 3 and 4, 5 and 6, separately; decayed and filled surfaces (D3-6MFS); number of decayed, filled and missing surfaces and teeth (D3-6MFS and D3-6MFT), as well as the significant caries index (SiC). To calculate the SiC index, participants were sorted according to their D3-6MFT values in descending order, and the D3-6MFT values of the top tertile were averaged (30).

### Statistical analysis

Descriptive statistics (e.g., mean, standard deviation, frequency, and percentages) were used to describe the study population. The normality of the distribution of continuous variables was tested using Shapiro–Wilk normality tests. In univariate analysis, comparisons between binary predictors, such as participants' relative weight status (overweight/obese vs. under-/healthy-weight), fruit and vegetable consumption and snacking habits (0–1/day vs. 2+/day), and biological sex (male/female) were done using Pearson's chi-square test for categorical outcomes, and Student's *t*-test or Wilcoxon rank-sum test for continuous outcomes, as appropriate.

In multivariable analysis, logistic regression models were used to estimate the odds ratios (ORs) and their 95% confidence intervals (95% CIs) for overall D3-6MFT and D3-6MFS indexes (yes/no) and untreated caries experience (yes/no) as outcomes, participants' relative weight as the exposure (using under/ healthy weight as the reference), while adjusting for potential confounders: age, sex, and dietary intake variables. All statistical tests were two-sided and conducted at a 0.05 level of statistical significance. All analyses were performed using the statistical software STATA version 15 (STATA Corp, College Station, TX, USA). Post-hoc power analysis was conducted using GPower statistical software, version 3.1. 9.6.

## Results

A total of 187 adolescents were enrolled in the study. Most of the adolescents were females (59.89%), and the mean age of the study population was 15.68 (±1.25) years (Table 1). The mean BMI percentile was 72.24 (±28.78); 48.66% of the adolescents were at a healthy weight; 19.79% were overweight, and 30.48% were obese. Nearly 68% of the participants had dental caries (D3-6MFT≥1); close to 46% of our study group had untreated caries. The overall mean D3-6MFT was 3.59 (±3.97), and D3-6MFS was 5.20 (±5.95), with the mean SiC estimated at 8.23 (±3.25). Additional descriptive statistics are depicted in Table 1.

**Table 1:** Descriptive statistics of the sample (*N* = 187).

Variable	Value
Sex, *n* (%)	
Female	112 (59.89)
Male	75 (40.11)
Age (years)	
Mean (SD)	15.68 (1.25)
Median (p25, p75)	16 (15, 17)
Range	13–20
BMI percentile	
Mean (SD)	72.24 (28.78)
Median (p25, p75)	85.2 (51.2, 96.7)
Range	3.36–99.71
Adjusted BMI, categorized, *n* (%)	
Underweight	2 (1.07)
Healthy weight	91 (48.66)
Overweight	37 (19.79)
Obese	57 (30.48)
Caries experience (D3-6MFT≥ ) (*n* = 170), *n* (%)	115 (67.65)
Untreated caries (D3-6T ≥1) (*n* = 170), *n* (%)	78 (45.93)
Number of carious surfaces per tooth with ICDAS scores 3 & 4 (*n* = 183)	
Mean (SD)	1.32 (2.13)
Median (p25, p75)	0 (0, 2)
Range	0–11
Number of carious surfaces per tooth with ICDAS scores 5 & 6 (*n* = 183)	
Mean (SD)	0.26 (1.10)
Median (p25, p75)	0 (0, 0)
Range	0–8
D3-6MFS (*n* = 183) (decayed and filled surfaces)	
Mean (SD)	5.17 (5.92)
Median (p25, p75)	3 (0, 8)
Range	0–36
D3-6T(*n* = 170) (decayed teeth)	
Mean (SD)	1.22 (1.88)
Median (p25, p75)	0 (0, 2)
Range	0–11
D3-6MFT (*n* = 170) (decayed, missing, and filled teeth)	
Mean (SD)	3.59 (3.97)
Median (p25, p75)	3 (0, 6)
Range	0–18
D3-6MFS (*n* = 183) (decayed, missing, and filled surfaces)	
Mean (SD)	5.20 (5.95)
Median (p25, p75)	3 (0, 8)
Range	0–36
SiC index (*n* = 57)	
Mean (SD)	8.23 (3.25)
Median (p25, p75)	7 (6, 10)
Range	4–18

*n*, frequency; %, percent; SD, standard deviation; p25 or p75, 25th or 75th percentile.

Adolescents in under- and healthy weight categories presented a higher caries prevalence (71.60% vs. 64.04%) and untreated caries prevalence (46.91% vs. 44.94%) as compared to adolescents in the overweight and obese categories. However, results were not statistically significant (*P *> 0.05). These results can be due to the small sample size and the higher number of participants in the healthy weight category. Differences in other caries indices (D3-6S and D3-6MFS) in two groups defined by overweight/obese status were not statistically significant (*P *> 0.05), with overweight/obese appearing to have somewhat higher averages for the D3-6S index, but lower estimates for D3-6MFS (Table 2). The SiC index estimates, however, were significantly higher in overweight/obese, mean SiC index for obese/overweight participants at 9.71 (±3.61)), and the mean for the healthy weight/underweight participants at 7.15 (±2.50) (*P *< 0.005) (Table 2). The mean D3-6MFT index for the population was 3.59 (±3.97).

**Table 2:** Distribution of caries indices, according to participants’ relative weight category.

Variable	Relative weight category		*p*-value
	Underweight/healthy weight (*N* = 93)	Overweight/obese (*N* = 94)	
Caries prevalence (D3-6MFT≥1), *n* (%)	*n* = 81	*n* = 89	0.293^a^
	58 (71.60)	57 (64.04)	
Untreated caries prevalence (D3-6T ≥1), *n* (%)	*n* = 81	*n* = 89	
	38 (46.91)	40 (44.94)	0.797^a^
D3-6S	*n* = 91	*n* = 93	0.817^b^
Mean (SD)	1.47 (2.16)	1.67 (2.98)	
Median (p25, 975)	0 (0, 3)	0 (0, 2)	
D3-6MFS	*n* = 90	*n* = 93	0.282^b^
Mean (SD)	5.30 (5.35)	5.04 (6.45)	
Median (p25, p75)	4 (0, 9)	3 (0, 8)	
SiC index	*n* = 33	*n* = 24	<0.005^b^
Mean (SD)	7.15 (2.50)	9.71 (3.61)	
Median (p25, p75)	7 (5, 8)	9.5 (7, 12)	
Range	4–15	4–18	

^a^ Pearson chi-square test, ^b^Wilcoxon rank-sum test.

Table 3 shows the distribution of self-reported dietary intake according to adolescents' BMI categories. Even though the differences were not statistically significant, a slight decrease in the proportion of fruit juice, snacks, and fruits consumption was observed among adolescents in the highest weight categories (overweight/obese) as compared to adolescents in the lowest weight categories (healthy weight/underweight) (*P *> 0.05). On the other hand, significantly higher consumption of soda, sports, and sugary drinks was reported among underweight/healthy weight adolescents as compared to their overweight/obese counterparts (38.89% of underweight/healthy weight adolescents reporting consuming 2–5 cups of soda, sports or sugary drinks per day vs. 22.58% of overweight/obese participants; *P* = 0.017). These results are based on a self-administered questionnaire that may have recall bias.

**Table 3:** Distribution of self-reported dietary intake, according to participants’ relative weight category.

Variable	BMI category		*p*-value^a^
	Underweight/healthy weight (*n* = 93)	Overweight/obese (*n* = 94)	
Fruit juice consumption, cups/day (*N* = 183)			0.759
0–1	62 (68.89)	66 (70.97)	
2–5	28 (31.11)	27 (29.03)	
Consumption of soda, sports, or sugary drinks, cups/day (*N* = 183**)**			0.017
0–1	55 (61.11)	72 (77.42)	
2–5	35 (38.89)	21 (22.58)	
Number of snacks (sweets, cookies, chips, etc.), consumed/day (*n* = 180)			0.469
0–1	45 (51.14)	52 (56.52)	
2–5	43 (48.86)	40 (43.48)	
Number of fruits (bananas, oranges, apples, grapes, etc.), consumed/day (*n* = 182)			0.482
0–1	69 (77.53)	76 (81.72)	
2–5	20 (22.47)	17 (18.28)	
Number of vegetables (green beans, broccoli, carrots, etc.) (*n* = 183)			0.774
0–1	75 (83.33)	76 (81.72)	
2–5	15 (16.67)	17 (18.28)	

^a^ Pearson's chi-square test.

No association was found between caries indices and participants' dietary intake of fruit juice, fruits, and vegetables consumed per day. Adolescents consuming 2–5 cups of soda or sugary drinks presented a significantly higher prevalence of caries (78.85% vs. 62.71%, chi-square *P *= 0.038) and untreated caries (57.69% vs. 40.68%, *P *= 0.04) than those reporting a lower consumption (≤1 portion). A significant association was observed between participants' consumption of snacks and the SiC index. Adolescents consuming 2–5 portions of snacks per day reported a higher median (p25, p75) SiC index of 8 (7, 12) as compared to those consuming ≤1 portion per day [median (p25, p75) SiC index: 7 (5, 9); *P* < 0.005] (Table 4).

**Table 4:** Association between caries indices and fruit, vegetable consumption, and snacking habits.

Variable	Fruit juice, cups/day		*p*-value
	0–1	2–5	
D3-6S	*n* = 128	*n* = 55	0.371^a^
Mean (SD)	1.41 (2.29)	1.91 (3.20)	
Median (p25, p75)	0 (0, 2)	1 (0, 3)	
D3-6MFS	*n* = 127	*n* = 54	0.607^a^
Mean (SD)	`4.86 (5.47)	5.76 (6.94)	
Median (p25, p 75)	3 (0, 8)	4 (0, 9)	
Caries prevalence (D3-6MFT≥1)	81 (67.50)	34 (68.00)	0.949^b^
Untreated caries prevalence (D3-6T ≥1)	53 (44.17)	25 (50.00)	0.487^b^
SiC index	*n* = 40	*n* = 17	0.080^c^
Mean (SD)	7.68 (3.07)	9.35 (3.64)	
Median (p25, p75)	7 (5.5, 10)	8 (7, 11)	
Variable	Soda or sugary drinks, cups/day		*p*-value
	0–1	2–5	
D3-6S	*n* = 127	*n* = 56	0.397^c^
Mean (SD)	1.45 (2.61)	1.80 (2.59)	
Median (p25, p75)	0 (0, 2)	1 (0, 2)	
D3-6MFS	*n* = 126	*n* = 55	0.108^c^
Mean (SD)	4.69 (6.05)	6.20 (5.59)	
Median (p25, p75)	3 (0,7)	5 (1, 10)	
Caries prevalence (D3-6MFT≥1)	74 (62.71)	41 (78.85)	0.038^b^
Untreated caries prevalence (D3-6T ≥1)	48 (40.68)	30 (57.69)	0.040^b^
SiC index	*n* = 39	*n* = 17	0.427^c^
Mean (SD)	7.85 (3.77)	8.65 (2.47)	
Median (p25, p75)	7 (5, 11)	8 (7, 10)	
Variable	Snacks, number/day		*p*-value
	0–1	2–5	
D3-6S	*n* = 97	*n* = 83	0.743^a^
Mean (SD)	1.34 (2.08)	1.82 (3.12)	
Median (p25, p75)	0 (0, 2)	0 (0, 3)	
D3-6MFS	*n* = 96	*n* = 82	0.245^a^
Mean (SD)	4.56 (5.38)	5.87 (6.56)	
Median (p25, p75)	3 (0, 7)	4 (0, 9)	
Caries prevalence (D3-6MFT≥1)	62 (67.39)	52 (68.42)	0.887^b^
Untreated caries prevalence (D3-6T ≥1)	43 (46.74)	34 (44.74)	0.795^b^
SiC index	*n* = 31	*n* = 26	0.036^a^
Mean (SD)	7.16 (2.61)	9.56 (3.36)	
Median (p25, p75)	7 (5, 9)	8 (7, 12)	
Variable	Fruits, number/day		*p*-value
	0–1	2–5	
D3-6S	*n* = 145	*n* = 37	0.721^a^
Mean (SD)	1.62 (2.74)	1.35 (2.00)	
Median (p25, p75)	0 (0, 2)	1 (0, 1)	
D3-6MFS	*n* = 143	*n* = 37	0.783^c^
Mean (SD)	5.17 (6.06)	4.86 (5.57)	
Median (p25, p75)	3 (0, 8)	3 (0, 8)	
Caries prevalence (D3-6MFT≥1)	91 (67.41)	23 (67.65)	0.979^b^
Untreated caries prevalence (D3-6T≥1)	60 (44.44)	18 (52.94)	0.374^b^
SiC index	*n* = 45	*n* = 11	0.334^c^
Mean (SD)	8.44 (3.39)	7.45 (2.73)	
Median (p25, p75)	7 (6, 11)	7 (5, 10)	
Variable	Vegetables, number/day		*p*-value
	0–1	2–5	
D3-6S	*n* = 151	*n* = 32	0.867^a^
Mean (SD)	1.64 (2.75)	1.19 (1.75)	
Median (p25, p75)	0 (0, 2)	0.5 (0, 2)	
D3-6MFS	*n* = 149	*n* = 32	0.294^c^
Mean (SD)	5.34 (5.92)	4.13 (6.00)	
Median (p25, p75)	4 (0, 8)	2 (0, 5)	
Caries prevalence (D3-6MFT≥1)	95 (68.35)	20 (64.52)	0.680^b^
Untreated caries prevalence (D3-6T≥1)	63 (45.32)	15 (48.39)	0.757^b^
SiC index	*n* = 46	*n* = 10	0.121^c^
Mean (SD)	8.52 (3.39)	7.30 (2.54)	
Median (p25, p75)	7.5 (6, 11)	7 (5, 9)	

^a^ Wilcoxon rank-sum test; ^b^Pearson's chi-square; ^c^Student's *t*-test.

In multivariable analysis, there was no statistically significant association between participants' BMI (in three categories, using healthy-/underweight as the reference) and overall caries experience as presented as D3-6MFT and D3-6MFS indexes as the outcome (Table 5). In the analysis for untreated caries experience as the outcome, overweight participants appeared to have somewhat higher odds of the outcome, compared to the under-/healthy weight, with odds ratio estimates ranging from 1.67 (95% CI: 0.74; 3.80) in the age- and sex-adjusted model to 1.83 (95% CI: 0.75; 3.92) obtained from the age-, sex- and soda consumption-adjusted model. On the other hand, the OR estimates for the obese category were close to null values (Table 6).

**Table 5:** Odds ratios (95% confidence intervals)^a^ for caries experience (D3-6MFT≥1, yes/no), according to participants’ relative weight.

Regression model	*N*	Overweight vs. healthy/underweight (ref.) OR (95% CI)^a^	Obese vs. Healthy/underweight (ref.) OR (95% CI)^a^
Model 1: BMI, age, and sex	170	0.83 (0.35; 2.01)	1.15 (0.51; 2.62)
Model 2: model 1+ fruit juice	170	0.84 (0.35; 2.02)	1.15 (0.50; 2.63)
Model 3: model 1+ soda/sugary drinks	170	0.90 (0.37; 2.19)	1.28 (0.55; 2.97)
Model 4: model 1+ snacks	168	0.84 (0.34; 2.05)	1.10 (0.48; 2.54)
Model 5: model 1+ fruits	169	0.88 (0.36; 2.14)	1.23 (0.53; 2.85)
Model 6: model 1+ vegetables	163	0.81 (0.33; 1.98)	1.25 (0.55; 2.87)

^a^ Estimates were obtained from logistic regression models with caries indexes D3-6MFT>1 (yes/no) as the outcome.

**Table 6:** Odds ratios (95% confidence intervals)^a^ for untreated caries experience (D3-6T≥1, yes/no), according to participants’ relative weight category.

Regression model	*N*	Overweight vs. healthy/underweight (ref.) OR (95% CI)^a^	Obese vs. healthy/underweight (ref.) OR (95% CI)^a^
Model 1: BMI, age, and sex	170	1.67 (0.74; 3.80)	0.92 (0.43; 1.98)
Model 2: model 1+ fruit juice	170	1.72 (0.75; 3.93)	0.87 (0.40; 1.91)
Model 3: model 1+ soda/sugary drinks	170	1.83 (0.79; 4.21)	1.01 (0.46; 2.22)
Model 4: model 1+ snacks	168	1.71 (0.75; 3.92)	0.88 (0.40; 1.92)
Model 5: model 1+ fruits	169	1.81 (0.79; 4.18)	0.97 (0.44; 2.14)
Model 6: model 1+ vegetables	170	1.68 (0.74; 3.83)	0.96 (0.44; 2.07)

^a^ Estimates were obtained from logistic regression models with untreated caries index D3-6T (yes/no) as the outcome.

## Discussion

This is the first study on caries and obesity in Puerto Rican adolescents between 13 and 19 years of age. We found a high prevalence of obesity and overweight in this group of Puerto Rican adolescents (50. 27% for obese and overweight, combined), like estimates reported by Elias et al. (8) among12-year-old schoolchildren in Puerto Rico, and Garza et al. (12) among a group of youth. However, caries experience or D3-6MFT and D3-6MFS was high and unrelated to the weight category in the adolescent population studied (*P* > 0.05). However, the severity of caries present by the adolescents in this study was found to be moderate. Based on ICDAS criteria findings, mean DS3-4 = 1.32 (2.13) or moderate decay had a greater mean index than severe decay index Mean DS5-6 = 0.26 (1.10) (Table 1).

Obesity and dental caries are worldwide problems that affect general health (1–3). The prevention of both dental caries and obesity has proven very difficult because these two diseases are multifactorial, and multiple strategies are required to deal with them (1, 2, 31). Both diseases demand behavioral changes that can be difficult for parents and adolescents (2, 31, 32). We hypothesized that we would find a positive association between BMI or obesity categories and dental caries experience as measured by D3-6MFT, D3-6MFS, D3-6T and D3-6S indexes in Puerto Rican adolescents aged 13–19 years of age from public and private schools. Still, study results provided no evidence of this association after adjusting for potential confounders. These results are due to the limitations of this study on sample size and power to detect differences. Recruitment problems of overweight and obese adolescents were an issue. Caries indexes were similar for healthy weight, overweight, and obese adolescents when the D3-6MFS was used for the whole population. However, when comparing the SiC index, significant differences were found between overweight/obese and healthy adolescents (*p* < 0.005). This SiC index finding points out the subgroups with more presence of disease: caries, restorations, and missing teeth (30). Our results were similar to those reported in other studies on obese adolescents (13–19). However, the finding of a high prevalence of overweight and obesity among the adolescent population studied should serve as an alert to Puerto Rico's public health policymakers that this issue must be addressed in future public health policy plans. The answers provided by the adolescents in our dietary assessment questionnaire, although a recall bias can exist, provided evidence of a possible association between high sugar intake and obesity, carbonated drink consumption, and caries severity level. The causes of caries and obesity diseases are quite complex (4, 5). The frequency of sugary foods and drinks could cause caries. Still, the exact quantity per portion of each type of food and drink is less relevant for caries than for overweight and obesity (1–3, 31). Future studies must focus on the differences between the risk factors for obesity and those of caries.

Limitations of the study include a relatively small sample size, especially for overweight, obese, and underweight categories. In our bivariate comparisons, we combined BMI categories to increase the statistical power of the tests, which precluded us from making more detailed comparisons. However, even with the combined BMI categories, the achieved *post hoc* statistical power for the bivariate analysis was estimated at around 6% for untreated caries prevalence, 18% for caries prevalence, 7% for D3-6S and 6% for D3-6MFS comparisons. In addition, our study was based on a convenience sample of adolescents from schools easily accessible to the study investigators, which may have affected the generalizability of our prevalence estimates. The study's cross-sectional nature does not allow for the establishment of temporality for the association between two chronic conditions of overweight/obesity and dental caries, closely linked to diet and other healthy behaviors. Despite these limitations, our study had several strengths. We used ICDAS criteria to assess caries, restorations and missing teeth due to caries and severity, and calibrated dental examiners conducted all assessments. Height and weight measurements were obtained by trained research staff, providing more accurate estimates and using a calibrated Tanita. Our analysis for the association was adjusted for dietary intake of sugary drinks, an established risk factor for dental caries and obesity. Nevertheless, the self-administered questionnaire did not ask about fluoride intake or exposure, the frequency of dental visits, or toothbrushing, which is a limitation when adjusting for confounding variables. However, we would like to state that there is no water fluoridation in Puerto Rico. Therefore, Puerto Rico public and private schools' students of all ages are requested to present a compulsory certificate each year at registration to attend the school from their dentist's providing information on dental exams, fluoride application, and prophylaxis. All toothpastes available at the market in Puerto Rico are fluoridated.

## Conclusions

Based on the limited number of participants, no association was found between dental caries (D3-6MFT and D3-6MFS indexes) and obesity (BMI) among adolescents. High rates of obesity were found in this study group.

## Data Availability

The raw data supporting the conclusions of this article will be made available by the authors, without undue reservation.
